# Thirty Years of Provident Dispensary Work
**The Light of Thirty Years of Provident Dispensary Work on National Insurance.* By a Provident Dispensary Medical Officer. (Manchester : Sherratt and Hughes.)


**Published:** 1911-08-19

**Authors:** 


### SPECIAL ARTICLE.

### THIRTY YEARS OF PROVIDENT DISPENSARY WORK.*

This may be described as a charming pamphlet in which the humanitarian, the medical, and the practical aspects of the work of Provident Medical Dispensaries are presented by a man of broad views and intelligent sympathies. To the medical man, possessed of a sensitive disposition, the troubles of the poor during illness or as the result of accident or disease, cannot fail to affect deeply the outlook upon life. With him medical work cannot be merely a means of livelihood. There are occasionally irritating, ungrateful, and inconsiderate people amongst patients, just as there are, no doubt, aggressive personalities amongst doctors. They both possess characters in which the ruling spirit is to get as much and give as little as possible. Happily, however, there are many to whom are given higher ideals or who are ruled by more generous impulses.

### A Plea for the Dispensary Patient.

The writer of this pamphlet is a man evidently blessed with generous impulses and who has found the spirit which animated the greater part of his poor patients has been the same. He says, "The members make it as easy for the doctor as they can commensurate with their needs, and are apologetic if they think they are troublesome," and further describes provident dispensary work to be "ideal and a delight in itself." The question of fees being out of sight "there is no barrier to hinder the access of the sick to the healer," and while from the patients' standpoint this absence of the money question removes a possible source of reserve, from the doctor's standpoint there is relief from a branch of work which he may find irksome. "He has no bookkeeping or other labour to waste his time or divert his thoughts from his proper work."

### Some Illustrations.

Illustrations are given of the struggles of the sick poor. One woman, a member of the dispensary for thirty years, had always to fight against incurable ill-health, "which was her undoing in the end, for struggle against it as she might, there was at last no result but the poor-house." On the same morning as this woman called to see the doctor a woman was seen whose husband was dying of consumption. There were six children, and after "necessary expenses" were paid (presumably house rent and expenses connected with the house) only a little over a shilling a week remained for the family to live upon. Again, another poor woman is referred to who lived by charing and never seemed to earn more than five shillings a week. "She was a good, brave woman, not without glimpses of the eternal." One day she was seen in the street peering curiously at a house in which the shutters were up in part. She asked if someone were dead there, and when answered that it was not known whether such were the case the woman stated with evident sincerity, "I wish it was me." This woman, who is described as "an upright soul to whom the joy of life had been denied," eventually entered the union.

### The Poor as the Dispensary Doctor Sees Them.

Short notes such as these give some insight into the life, it is to be feared, of a considerable portion of the community whose lot everyone possessing any feeling of sympathy with his fellow-men would like to see improved. Such a desire must have been present in many minds before and since the days of Defoe, who asked his fellow-countrymen more than two hundred years ago "to consider the present state of this kingdom," and suggested insurance for the sick and those out of employment, expressing the hope that by some such means it might be possible "for ever to banish beggary and poverty out of the kingdom."

### The Business Side of the Work.

To leave, however, the humanitarian and speak of the business aspects of a provident dispensary, the writer of this pamphlet soon arrived at the conclusion that a doctor could not live upon the money he received from work in a dispensary unless he were to adopt a life of celibacy. The work could be combined with more lucrative private practice, but allowing that, by continuing the two kinds of practice, a moderate income might be obtained, the fact would remain that services given to a dispensary would in a certain measure be under such circumstances the services of charity. Apparently, those responsible for the management of the dispensary did not look at the matter in this light. The fact that there were charities in the City for attendance at which doctors received nothing, while at the dispensary payment was given for services, was sufficient ground for the committee to consider that the doctors should be thankful for small mercies. Eventually there was conflict between the medical staff and lay committee, which resulted in the members ranging themselves in a very decided manner on the side of the doctors. The lay committee then resigned and the doctors took the management into their own hands. This occurrence is mentioned as an illustration of the "eager readiness of patients to entertain grateful feeling towards their doctors. Earn their confidence and they become your faithful adherents." To refer to monetary details it may be mentioned that during twenty-eight years the capitation fee received by the doctors has averaged 3s. 1d. and the amount received per attendance 7.774 pence.

### The Effect of the Insurance Bill.

Views are expressed in this pamphlet upon various aspects of National Insurance from the medical standpoint. One is "the crying need of women and children for the doctor's healing help."

* *The Light of Thirty Years of Provident Dispensary Work on National Insurance.* By a Provident Dispensary Medical Officer. (Manchester: Sherratt and Hughes.)

It is stated: "Not only have mothers more need of the doctors' services for themselves than the fathers have, but also their need is perhaps more urgent still touching the children." With regard to the manner in which doctors are to be paid, it is considered, no doubt correctly, that payment per visit would add to the difficulties of the doctor's work on account of the amount of clerical work it would involve, and the authorities would need to check the doctor's statements by some method of inspection, which would add to the cost of administration. Again, if paid by the case, the more illness the better for the doctor; if paid by head, the less the better. In the latter case self-interest causes a leaning towards the preventive side of medicine. Further, the opinion is expressed that some doctors fear contract practice because they seem to think that they will be victimised by unreasonable patients. This is not the writer's experience. He states that patients "do not willingly give trouble, but are anxious to make the doctor's work as easy as may be." With regard to the capitation fee, it is remarked that if the doctors are to engage cheerfully in the work they must be satisfied. If dissatisfied the work will not be efficient. The calculation is made that a visiting fee of 2s. 6d. would require a capitation fee of 12s. After taking off 2s. for medicine this would leave 10s. for the doctor.

Although the writer of the pamphlet considers that doctors will not do the work heartily under a capitation fee of 10s. he seems to think that some National Insurance scheme will sooner or later be in operation, and adds that "the future is full of promise for the healing of the sick." The opinion is expressed that when free access is given to doctors to all who need them that "the unregulated pretensions of quacks will vanish." Evidently the writer is deeply in sympathy with the scheme, but here, as nearly everything that concerns human nature, the question of money looms large. Yet it is stated "the doctor is not mercenary," and, speaking generally, this is no doubt true. But there is a genuine feeling that he is going to suffer, and that his income is to be seriously affected by the law of the land naturally disturbs his peace of mind.

